# A cluster-randomized trial to reduce caesarean delivery rates in Quebec: cost-effectiveness analysis

**DOI:** 10.1186/s12916-017-0859-8

**Published:** 2017-05-22

**Authors:** Mira Johri, Edmond S. W. Ng, Clara Bermudez-Tamayo, Jeffrey S. Hoch, Thierry Ducruet, Nils Chaillet

**Affiliations:** 1 Centre de Recherche du Centre Hospitalier de l’Université de Montréal (CRCHUM), Tour Saint-Antoine, Porte S03-910, 850, rue St-Denis, Montréal, Québec H2X 0A9 Canada; 2 Department of Health Management, Evaluation and Policy, School of Public Health, University of Montreal, Montréal, Québec Canada; 3 Department of Maternal, Neonatal, Child and Adolescent Health, World Health Organization, Geneva, Switzerland; 4 Director’s Office, London School of Hygiene and Tropical Medicine (LSHTM), London, UK; 5 Department of Obstetrics and Gynecology, Centre Hospitalier Universitaire (CHU) de Sherbrooke, Sherbrooke, Québec Canada; 6 Andalusian School of Public Health, Granada, Spain; 7 CIBER Epidemiologia y Salud Publica (CIBERESP), Instituto de Salud Carlos III, Madrid, Spain; 8 Institute of Health Policy, Management and Evaluation, University of Toronto, Toronto, Ontario Canada; 9 Centre for Excellence in Economic Analysis and Research (CLEAR), Li Ka Shing Knowledge Institute, St Michael’s Hospital, Toronto, Ontario Canada; 10 Department of Public Health Sciences, University of California, Davis, California USA; 11 Department of Biostatistics, Centre hospitalier universitaire (CHU) Sainte-Justine, Université de Montréal, Montréal, Québec Canada; 12 Département Obstétrique et Gynécologie, Centre Hospitalier de l’Université Laval (CHUL), Québec, Québec Canada

**Keywords:** Randomized controlled trial, Cost-benefit analysis, Caesarean section/utilization, Pregnancy outcomes, Medical audit, Guideline adherence, Multilevel analysis, Female, Adult, Adolescent, Infant, Newborn

## Abstract

**Background:**

Widespread increases in caesarean section (CS) rates have sparked concerns about risks to mothers and infants and rising healthcare costs. A multicentre, two-arm, cluster-randomized trial in Quebec, Canada assessed whether an audit and feedback intervention targeting health professionals would reduce CS rates for pregnant women compared to usual care, and concluded that it reduced CS rates without adverse effects on maternal or neonatal health. The effect was statistically significant but clinically small. We assessed cost-effectiveness to inform scale-up decisions.

**Methods:**

A prospective economic evaluation was undertaken using individual patient data from the Quality of Care, Obstetrics Risk Management, and Mode of Delivery (QUARISMA) trial (April 2008 to October 2011). Analyses took a healthcare payer perspective. The time horizon captured hospital-based costs and clinical events for mothers and neonates from labour onset to 3 months postpartum. Resource use was identified and measured from patient charts and valued using standardized government sources. We estimated the changes in CS rates and costs for the intervention group (versus controls) between the baseline and post-intervention periods. We examined heterogeneity between clinical subgroups of high-risk versus low-risk pregnancies and estimated the joint uncertainty in cost-effectiveness over 20,000 trial simulations. We decomposed costs to identify drivers of change.

**Results:**

The intervention group experienced per-patient reductions of 0.005 CS (95% confidence interval (CI): −0.015 to 0.004, *P* = 0.09) and $180 (95% CI: −$277 to − $83, *P* < 0.001). Women with low-risk pregnancies experienced statistically significant reductions in CS rates and costs; changes for the high-risk subgroup were not significant. The intervention was “dominant” (effective in reducing CS and less costly than usual care) in 86.08% of simulations. It reduced costs in 99.99% of simulations. Cost reductions were driven by lower rates of neonatal complications in the intervention group (−$190, 95% CI: −$255 to − $125, *P* < 0.001). Given 88,000 annual provincial births, a similar intervention could save $15.8 million (range: $7.3 to $24.4 million) in Quebec annually.

**Conclusions:**

From a healthcare payer perspective, a multifaceted intervention involving audits and feedback resulted in a small reduction in caesarean deliveries and important cost savings. Cost reductions are consistent with improved quality of care in intervention group hospitals.

**Trial registration:**

International Clinical Trials Registry Platform, 10.1186/ISRCTN95086407. Registered on 23 October 2007

**Electronic supplementary material:**

The online version of this article (doi:10.1186/s12916-017-0859-8) contains supplementary material, which is available to authorized users.

## Background

The use of caesarean sections has increased to unprecedented levels worldwide; 18.6% of global and 32.3% of North American births now occur by caesarean section [1]. With the exception of the African region, where caesarean section rates remain low, substantial increases in caesarean deliveries have been documented in all global regions [1]. Caesarean sections are important in reducing maternal and perinatal mortality and morbidity when medically justified; however, non-medically necessary caesareans have no documented benefit [2]. In many jurisdictions, caesarean section rates in excess of recommended thresholds have sparked concerns about potential risks to mothers and infants and escalating healthcare costs [1, 3, 4]. Evidence concerning effective approaches to reduce unnecessary caesareans is currently limited [3, 5].

The Quality of Care, Obstetrics Risk Management, and Mode of Delivery (QUARISMA) study was a cluster-randomized controlled trial conducted from 2008 to 2011 in 32 hospitals in Quebec, Canada [6]. The trial assessed whether a multifaceted audit and feedback intervention targeting health professionals involved in labour, delivery, and postpartum care would reduce caesarean section rates as compared to usual care for pregnant women [6]. The idea motivating the QUARISMA trial was that an effective knowledge translation intervention could improve adherence to clinical practice guidelines and quality of care, thereby reducing unnecessary caesareans and optimizing resource use.

The main trial analysis found a statistically significant but clinically small reduction in caesarean sections (adjusted absolute risk difference of −1.8% (95% CI: −3.8 to −0.2, *P* = 0.04) with no adverse effects on maternal or neonatal health outcomes [6]. The reduction in caesareans was observed among women with low-risk pregnancies but not among women with high-risk pregnancies [6]. Although resources required to deliver the intervention are modest, the anticipated magnitude of clinical benefit is small, leaving unanswered questions about whether the intervention should be offered at scale. A cost-effectiveness analysis is required to complete information from the trial and enable decision makers to interpret results for policy and practice.

We conducted a cost-effectiveness analysis of the QUARISMA trial using individual patient data. Our primary objective was to compare the impact on caesarean delivery rates and costs of a multifaceted audit and feedback intervention targeting health professionals versus usual care for pregnant women. Subgroup analyses examined cost-effectiveness in high-risk versus low-risk pregnancies, the clinical subgroups established a priori for the trial.

## Methods

The QUARISMA trial was conducted in the province of Quebec, Canada. In 2014, Quebec’s population was 8.2 million, and life expectancy at birth was 79 for males and 83 for females [7]. Gross domestic product per capita measured in US dollar purchasing power parity was $36,216 in 2013 [8]. The QUARISMA economic evaluation adopted the perspective of the publicly funded healthcare system in Quebec. Analysis of effects focused on the primary hypothesized trial outcome of reduction in caesarean sections, while cost analysis examined direct costs to the public healthcare system. Cost variables capture the full spectrum of resource use related to labour and delivery from hospital admission for labour until 3 months postpartum. As delivery mode (caesarean or vaginal) was the primary trial endpoint, a short-term hospital stay is sufficient to capture all meaningful differences in costs and clinical outcomes between intervention and control arms. Costs and health outcomes were discounted at an annual rate of 0% due to the short (<1 year) period for results assessment [9, 10].

All participating hospital centres granted research ethics approval for the trial.

### Trial overview

Details of the QUARISMA trial have been reported [6]. Briefly, QUARISMA employed a cluster-randomized design with a 1:1 allocation ratio. Clusters were 32 public hospitals in the province of Quebec, Canada. Hospitals were stratified by level of care and assigned to either the intervention or control group using computer-generated blocked randomization within each stratum. Hospital centres were eligible to participate if they had at least 300 deliveries in the year prior to study initiation, a caesarean section rate of at least 17%, and no concurrent program to reduce caesareans. Based on these criteria, 40 hospitals were eligible, 38 agreed to participate, and 32 were randomly selected for inclusion. All women who gave birth at a participating hospital during the study period and whose newborns met criteria related to gestational age (≥24 weeks) and birth weight (≥500 g) were included in the analysis.

The study comprised three phases: a 1-year pre-intervention (baseline) period, a 1.5-year intervention period, and a 1-year post-intervention period. The baseline period involved onsite training and capacity building to improve caesarean delivery and intrapartum care. During the 1.5-year intervention period, hospital audit committees implemented four 3-month audit cycles using local data to assess the appropriateness of caesarean delivery, engage in collective learning, provide feedback to clinicians, and implement best practices based on the results. Analyses for the main trial and the economic evaluation compared outcomes in the 1-year baseline period (1 April 2008 to 31 March 2009) to those in the 1-year post-intervention period (1 November 2010 to 31 October 2011). The QUARISMA trial captured more than 65% of all deliveries in Quebec province during the study period [11]. No hospital or woman was lost to follow-up.

### Sample size

QUARISMA was designed and powered to detect differences between treatment groups in the primary clinical endpoint (caesarean sections averted). Sample size calculations were not designed to test cost-effectiveness hypotheses.

### Effects

We recorded clinical events from patient charts. Trained data collectors extracted data concerning caesarean or vaginal delivery as well as secondary outcomes including major and minor maternal complications and major and minor neonatal complications [6]. Trained research nurses or medical archivists abstracted in-hospital data from the medical records of mothers and newborns 3 months after delivery. Data collectors were aware of randomization assignments but were not involved in outcomes assessment. Given the nature of the clinical condition and the short time horizon, we did not include preference-based measures of health-related quality of life.

### Resource use and costs

We considered resource use and costs associated with delivery and complications recorded in the QUARISMA trial (Additional file 1: Section 3; Table S1) [6]. Medical procedure costs (Additional file 1: Table S2 and Table S3) were calculated as the sum of inpatient (hospital) costs and physician fees. For all trial participants contributing data to either the baseline or post-intervention period, we used inpatient chart data to identify clinical events generating resource use. To estimate inpatient procedure costs, we applied unit costs from the 2013 Canadian Institute for Health Information (CIHI) Patient Cost Estimator (PCE) for the jurisdiction of Quebec to resource use categories [12]. Published annually by the Canadian government, PCE costs represent the product of the costs of a standard hospital stay in a specific jurisdiction multiplied by a resource intensity weight reflecting resource use for a specific case mix and age group [12]. The 2013 PCE release includes 2010–2011 financial information [12], which corresponds to the QUARISMA post-intervention period. PCE costs exclude physician fees, which were taken from Canada’s National Physician Database (NPD), 2011–2012 [13]. Total costs also included resource requirements and costs associated with delivery of the QUARISMA intervention (Additional file 1: Table S4), which were estimated directly from QUARISMA trial records. Protocol-driven costs were excluded [9]. All costs are reported in 2013 Canadian dollars. In 2013, one US dollar was worth on average 1.03 Canadian dollars [14].

### Statistical analysis

Analyses for the QUARISMA economic evaluation were based on the intention-to-treat principle and carried forward strategies from the main trial analysis [6]. We adopted a variant of the difference-in-differences approach to estimate the intervention effect while controlling for unobserved characteristics of individual patients or program placement in hospitals that might lead to selection bias [15]. Specifically, to assess intervention impact, we studied changes in caesarean delivery rates and costs in the two study groups between the 1-year baseline period and the 1-year post-intervention period using adjusted regression coefficients (with their 95% confidence intervals) for the interaction between group (intervention or control) and time period (post-intervention or baseline) [6, 16].

#### Main analysis

We modelled costs and effects jointly using bivariate multilevel linear models that explicitly recognize potential correlations between the bivariate outcomes (caesarean sections and costs) at individual and cluster (hospital) levels. In our models, individual patients (level 1) were assumed to be nested within hospitals (level 2), and correlations were estimated at both hierarchical levels. We used cluster-level random effects to handle clustering of observations within hospitals [17, 18]. The analysis included all trial participants. Crude models studied the intervention effect by estimating the interaction between study group and time period. Adjusted models estimated the same interaction term while including additional baseline covariates (pregnancy risk, parity, current smoking, birth weight, and hospital type) used in the QUARISMA trial analysis [6]. Models were estimated by restricted unbiased iterative generalized least-squares estimation implemented in MLwiN (version 2.35) [19] within Stata 14.1 using the *runmlwin* [20] command.

We had intended to calculate the incremental cost-effectiveness ratio (ICER) statistic as the ratio of additional costs per additional caesarean sections averted. However, interpretation is problematic when ICER estimates are negative, as in this case where the intervention reduces costs [21]. Incremental costs and effects are therefore presented separately.

#### Uncertainty and heterogeneity of the cost-effectiveness measures

We applied Bayesian Markov chain Monte Carlo methods to the bivariate multilevel linear models used in the main analysis to ascertain the joint uncertainty of the estimands, incremental costs, and effects [22]. The posterior distributions of the incremental costs and effects were given by 20,000 iterations of the Markov chains after a burn-in of 5000 iterations. We used cost-effectiveness planes plotting the values of incremental costs and effects stored in the Markov chains to present the joint uncertainty of the estimands. To explore potential heterogeneity in the results, we prospectively planned to repeat all analyses over patient risk subgroups [21].

#### Other analyses

We adapted the bivariate multilevel linear models from the main analysis to consider two further issues. (1) NPD estimates for physician billings are uncertain [13] and can substantially influence total costs. We prospectively planned sensitivity analyses using alternative physician fee estimates from the Quebec medical specialists billing manual [23]. (2) To gain insight into cost drivers, we decomposed total costs into costs associated with delivery, maternal complications, and neonatal complications. Costs were grouped into these three categories because statistical power was insufficient to examine cost differences on a per-complication basis. Analyses of cost components were empirically motivated.

An independent team not involved in the main trial analysis conducted the economic analysis. All reported *P* values are two-sided. *P* values lower than 0.05 were considered statistically significant. Additional file 1: Section 2 expands on the statistical analysis; Tables S8 and S9 describe data clustering.

## Results

### Participants

All (100%) of the 105,351 women who delivered in the baseline or post-intervention period were included in the QUARISMA main trial analysis [6] and the economic evaluation. Analysis of baseline hospital, cost, and patient characteristics (Additional file 1: Table S5) revealed no significant differences between study groups, with the exception of maternal parity, which was included as a covariate in the adjusted analyses.

### Incremental costs and effects

Table 1 presents main analysis results concerning the incremental change in effects and costs due to the QUARISMA intervention. Analyses including all patients showed a small non-significant reduction in caesareans in the intervention group as compared to controls and an important reduction in costs, yielding adjusted estimates (Table 1, Model 2) of a per-patient reduction of 0.005 caesarean sections (95% confidence interval (CI): –0.015 to 0.004, *P* = 0.09) and $180 saved (95% CI: −$277 to − $83, *P* < 0.001). Patterns differed by patient risk. Women with low-risk pregnancies experienced larger, statistically significant reductions in caesarean sections and costs. Women with high-risk pregnancies experienced a small, non-significant increase in caesarean sections and a non-significant reduction in costs. Sensitivity analyses using alternative cost estimates predicted lower estimated savings but similar conclusions (Additional file 1: Table S6).

**Table 1:** Impact of the QUARISMA intervention on caesarean sections and total direct medical costs^a, b^

	Model 1: BMLM (crude)				Model 2: BMLM (adjusted)			
	Coef. (ß)	Std. Err.	95% CI	*P* value	Coef. (ß)	Std. Err.	95% CI	*P* value
All participants^c^ (*N* = 105,351 patients; 32 hospitals)^c^								
Effects (CS)	−0.009	0.005	(−0.019 to 0.002)	0.096	−0.005	0.005	(−0.015 to 0.004)	0.288
Costs ($)	−185	50	(−283 to −86)	<0.001	−180	49	(−277 to −83)	<0.001
Low-risk subgroup^d^ (*N* = 49,281 patients; 32 hospitals)								
Effects (CS)	−0.013	0.005	(−0.023 to −0.003)	0.013	−0.014	0.005	(−0.024 to −0.004)	0.005
Costs ($)	−210	63	(−334 to −87)	0.001	−226	62	(−348 to −105)	<0.001
High-risk subgroup^d^ (*N* = 56,070 patients; 32 hospitals)								
Effects (CS)	0.009	0.008	(−0.007 to 0.024)	0.291	0.008	0.008	(−0.008 to 0.024)	0.307
Costs ($)	−102	76	(−250 to 47)	0.180	−97	75	(−243 to 49)	0.193

*BMLM* bivariate multilevel linear model, *CS* caesarean section

^a^All costs given in 2013 Canadian dollars = (0.94 USD) [30]

^b^Total costs calculated using CIHI National Physician Database 2011–2012 physician fees for the Province of Quebec [13]

^c^Adjusted models for all participants included the following covariates: parity, smoking, birth weight, hospital type, and women’s risk level

^d^Adjusted subgroup models included the following covariates: parity, smoking, birth weight, and hospital type

### Uncertainty of incremental costs and effects

**Fig. 1:**
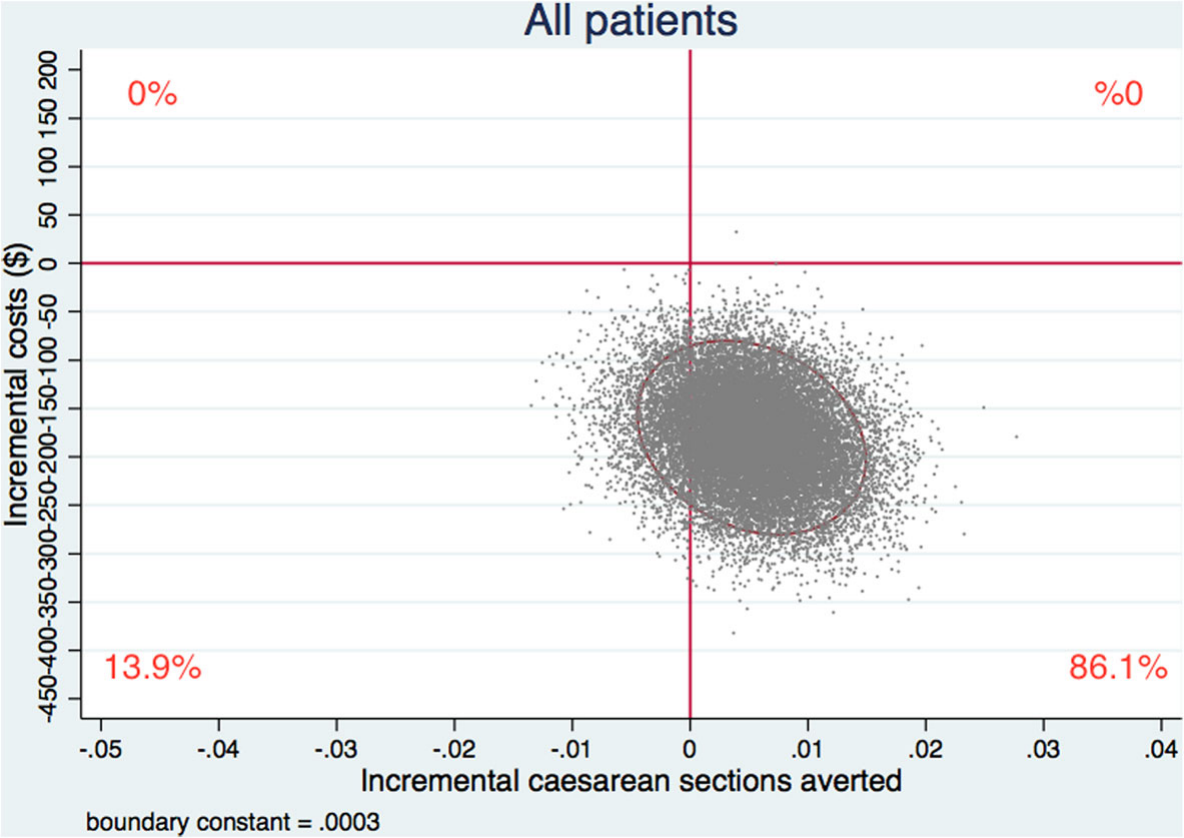
Incremental cost-effectiveness of the QUARISMA intervention versus routine care. Cost-effectiveness (*CE*) plane for the covariate-adjusted cost-effectiveness analysis of the QUARISMA intervention versus routine care. Incremental cost-effectiveness results were based on 20,000 Markov chain Monte Carlo iterations including all trial participants (*N* = 105,351). An ellipse containing 95% of the joint posterior distribution of incremental costs and effects is used to represent uncertainty on the CE plane. The centre of the ellipse represents the point estimate of incremental effects and costs, i.e. a per-patient reduction of 0.005 caesarean sections and $180 saved. Percentages represent the distribution of points by quadrant

**Fig. 2:**
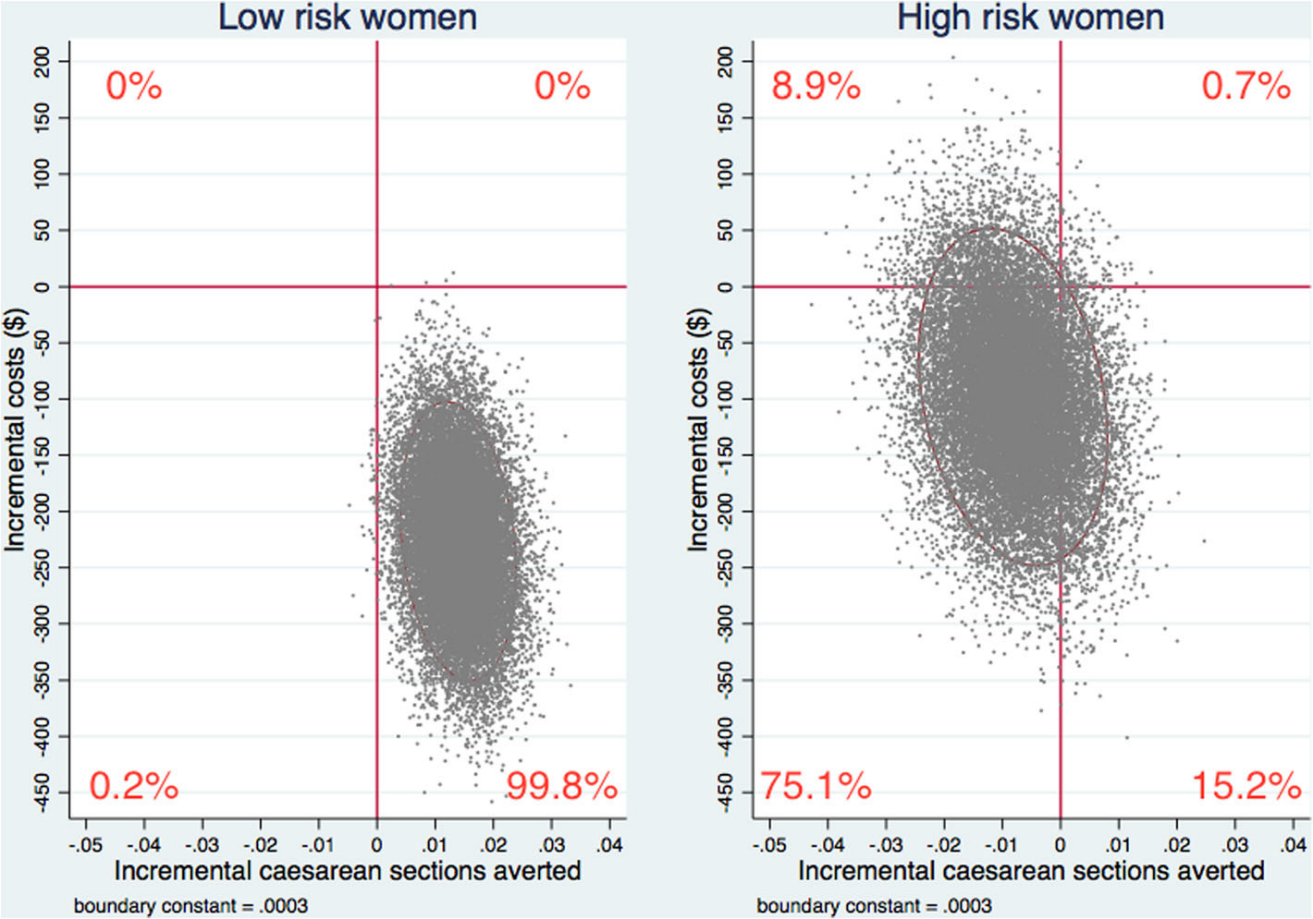
Incremental cost-effectiveness of the QUARISMA intervention versus routine care, by risk subgroups. Cost-effectiveness (*CE*) planes for the covariate-adjusted cost-effectiveness analysis of the QUARISMA intervention versus routine care, by patient risk subgroups. Incremental cost-effectiveness results were based on 20,000 Markov chain Monte Carlo iterations estimated separately for low risk (*N* = 49,281) and high-risk (*N* = 56,070) trial participants. An ellipse containing 95% of the joint posterior distribution of incremental costs and effects is used to represent uncertainty on the CE plane. The centre of the ellipse represents the point estimate of incremental effects and costs, i.e. a per-patient reduction of 0.014 caesarean sections and $226 saved for the low-risk subgroup, and a per-patient increase of 0.008 caesarean sections and $75 expenditure for the high-risk subgroup. Percentages represent the distribution of points by quadrant; figures may not sum to 100 due to rounding

Figures 1 and 2 present the joint uncertainties in cost and effectiveness estimates from the Bayesian Markov chain Monte Carlo analyses on the cost-effectiveness plane. Analyses including all patients (Fig. 1) revealed that the intervention reduced caesarean sections and costs in 86.01% of the Monte Carlo iterations, and reduced costs in 99.99% of iterations. For women with low-risk (high-risk) pregnancies (Fig. 2), the intervention reduced caesarean sections in 99.81% (15.97%) of the iterations, reduced costs in 99.98% (90.36%), and reduced both outcomes in 99.79% (15.24%). Sensitivity analyses using alternative cost estimates generated similar results (Additional file 1: Figure S1 and Figure S2).

### Cost subcategories

Table 2 presents changes in per-patient costs by subcategories of delivery costs, maternal complications, and neonatal complications. Findings suggest that the intervention to reduce caesarean sections resulted in improved quality along the continuum of care. Adjusted analyses including all patients revealed that the major driver of cost reductions was management of neonatal complications (−$190, 95% CI: −$255 to − $125, *P* < 0.001), which were less frequent in the intervention group [6]. Reductions in management costs for neonatal cardiopulmonary complications were especially important (−$150, 95% CI: −$197 to − $103, *P* < 0.001); costs for other neonatal complications were also reduced (−$41, 95% CI: −$80 to − $1, *P* < 0.047). Results using alternative cost assumptions were similar (Additional file 1: Table S7).

**Table 2:** Changes in per-patient costs due to the intervention, by clinical category^a, b^

Cost component	BMLM (crude)				BMLM (adjusted)			
	Coef. (ß) ($)	Std. Err. ($)	95% CI ($)	*P* value	Coef. (ß) ($)	Std. Err. ($)	95% CI ($)	*P* value^c^
All participants (*N* = 105,351 patients; 32 hospitals)								
Delivery (caesarean or vaginal)^d^	−8.3	8.2	(−24.4 to 7.7)	0.310	−6.2	7.7	(−21.3 to 9.0)	0.424
Maternal complications^e^	19.9	30.0	(−38.9 to 78.8)	0.507	16.3	29.9	(−42.3 to 74.8)	0.586
Neonatal complications^f^	−196	33.8	(−262 to −130)	<0.001	−190	33.3	(−255 to −125)	<0.001
Low-risk group (*N* = 49,281 patients; 32 hospitals)								
Delivery (caesarean or vaginal)^d^	−17.2	9.4	(−35.7 to 1.3)	0.069	−22.0	9.0	(−39.5 to −4.5)	0.014
Maternal complications^e^	22.0	38.4	(−53.2 to 97.2)	0.566	14.7	38.1	(−59.9 to 89.3)	0.700
Neonatal complications^f^	−215	42.0	(−297 to −133)	<0.001	−219	41.8	(−301 to −137)	<0.001
High-risk group (*N* = 56,070 patients; 32 hospitals)								
Delivery (caesarean or vaginal)^d^	17.6	12.2	(−6.3 to 41.6)	0.149	14.8	12.2	(−9.0 to 38.7)	0.223
Maternal complications^e^	17.6	45.3	(−71.2 to 106)	0.698	14.2	45.1	(−74.3 to 103)	0.753
Neonatal complications^f^	−136	51.2	(−237 to −36.0)	0.008	−126	50.0	(−224 to −27.6)	0.012

*BMLM* bivariate multilevel linear model, *CI* confidence interval

^a^Model coefficients (ß), standard errors, and 95% confidence intervals present costs given in 2013 Canadian dollars = (0.94 USD) [30]

^b^Total costs calculated using CIHI National Physician Database 2011–2012 physician fees for the Province of Quebec [13]

^c^The *P* value is the probability of observing a result at least as extreme as the z-statistic, for the null hypothesis that the difference in the rate changes in costs for the intervention group from baseline to post-intervention versus the control group from baseline to post-intervention does not differ from zero

^d^Delivery costs include: caesarean delivery (primary or secondary, with or without induction, with or without uterine scar, pediatric or adult); vaginal delivery (with or without anaesthetic, with or without intervention, assisted or unassisted, pediatric or adult)

^e^Maternal complication costs include: maternal death, hysterectomy, symptomatic uterine rupture, thromboembolic disease, internal organ injuries, perineal tear (grades 3–4), puerperal infection/sepsis, postpartum hospital stay ≥7 days, admission to intensive care unit, readmission to hospital after postpartum discharge, blood transfusion

^f^Neonatal complication costs include: intrapartum/neonatal death, Apgar score (<4; 4–7), major and minor acidosis (pH <7; pH 7–7.1), major and minor trauma, intraventricular haemorrhage, seizure at less than 24 h, invasive and non-invasive mechanical ventilation, necrotizing enterocolitis, hypoxic-ischaemic encephalopathy, cardiopulmonary morbidity, neonatal infection/sepsis, blood transfusion

## Discussion

The QUARISMA trial found that an intervention involving clinical audits, feedback, and implementation of best practices resulted in a statistically significant but small reduction in caesarean sections without adverse effects on maternal or neonatal outcomes [6]. In this economic evaluation conducted alongside the main trial, the analysis including all trial participants found that the intervention also conferred significant average cost savings of $180 (95% CI: $83–277) per patient, equivalent to 3.0% (95% CI: 1.4–4.5%) of mean per-patient total costs. Patterns differed by patient risk. Women with low-risk pregnancies experienced statistically significant reductions in caesarean sections and costs, while women with high-risk pregnancies did not experience significant changes in either outcome.

Sensitivity analyses exploring uncertainty in cost-effectiveness estimates over 20,000 trial simulations demonstrated that QUARISMA was the “dominant” intervention (effective in reducing caesarean sections and less costly than usual care) 86.08% of the time. The intervention reduced costs 99.99% of the time. Cost savings largely reflect the reduced costs of managing neonatal complications, which occurred less frequently in the intervention group [6].

Our findings are important for clinicians and policymakers interested in the care of pregnant women and neonates. The QUARISMA trial tested a multifaceted strategy involving audits and feedback to enable groups of health professionals to improve the quality of labour and delivery care at participating hospitals. In addition to achieving a statistically significant but clinically small benefit in the primary outcome of reducing caesarean sections, QUARISMA also demonstrated benefits for a variety of secondary clinical endpoints consistent with improvements in the standard of care implemented in intervention group hospitals [6]. Secondary trial outcomes revealing statistically significant improvements included minor neonatal morbidity (i.e. cardiopulmonary morbidity, moderate acidosis (pH ≥7 and <7.1), minor trauma, and neonatal infection/sepsis) and major neonatal morbidity (i.e. major trauma, use of invasive mechanical ventilation, and intrapartum and neonatal deaths) [6]. Economic evaluation offers a framework within which complex changes can be synthesized to aid in policymaking. Our findings (Table 1) suggest that if a similar intervention were to be delivered at scale in the Province of Quebec, for an annual provincial birth cohort of approximately 88,000 [11] and a per-patient reduction in caesareans of 0.005 (95% CI: −0.015 to 0.004), one could anticipate a reduction of roughly 440 (1320 to 0) caesarean sections. For the same annual birth cohort of 88,000 [11] and a per-patient reduction in costs of $180 (95% CI: −$277 to − $83), this translates to a cost savings of $15.8 million (range: $7.3 to $24.4 million), achieved without increasing maternal and neonatal morbidity and mortality.

These results have important implications for the scientific literatures evaluating audit and feedback approaches for quality improvement and non-clinical interventions to reduce caesarean sections. The audit and feedback strategy is widely used to promote quality improvement. A recent Cochrane systematic review found that audit and feedback generally leads to small but potentially important improvements in professional practice [24], which coheres well with the QUARISMA trial finding that the intervention conferred a modest reduction in caesarean sections [6]. Intervention impact on healthcare costs is often an outcome of central interest for quality improvement interventions. Yet, we found only one economic evaluation of an audit and feedback intervention based on data from a randomized trial, published by Fretheim and colleagues in 2006 [25]. This study is a well-conducted evaluation of a small, individually randomized trial in Norway, which found potential cost savings due to the intervention [25]. Analytic methods have advanced considerably in the interim; notably, the Fretheim and colleagues study was not based on individual patient data.

Important studies of non-clinical interventions to reduce unnecessary caesareans exist [5]; however, only one has published an economic evaluation [26]. Hollinghurst and colleagues conducted a cost-consequences analysis of a small, individually randomized trial of 742 pregnant women in the UK with a prior caesarean section [26], to evaluate two computer-based decision aids to reduce repeat caesarean sections [27]. One decision aid reduced repeat caesareans and was likely cost-neutral [27].

Ours is the first economic evaluation of a non-clinical intervention to reduce caesarean section rates based on a cluster-randomized trial. The QUARISMA trial was well designed and implemented and captured 65% of provincial births occurring in the trial period, plausibly representing the general population of pregnant women in Quebec province. Prospective data collection improved data completeness, accuracy, and coherence, while the use of standardized government sources for costing enhanced generalizability. The economic analysis was based on the intention-to-treat principle; it used complete data from individual patients and appropriate statistical methods that accounted for clustering and correlated outcomes to derive methodologically robust estimates of between-group differences in costs and effects.

Several limitations should be considered. (1) We assigned unit costs using Canadian government estimates computed annually through established methodologies. Because physician payment mechanisms in Canada are heterogeneous, physician cost estimates are less reliable than those for inpatient costs [13]. We repeated all analyses using an alternative data source representing billable fees per procedure, which provides a lower bound for physician costs and conservatively estimates cost savings. (2) The economic evaluation used a linear model to study mean costs and effects. This enabled us to address the key policymaking question of interest to decision makers, which concerns the absolute change in effects per change in costs conferred by the intervention. Linear regression models may result in incorrect standard errors. While our bivariate multilevel linear models account for clustering and bivariate correlations at hospital and individual levels, the use of Markov chain Monte Carlo methods to generate joint posterior distributions of incremental costs and effects yielded sample means and estimates of uncertainty robust to skewed costs [17, 28]. The main trial analysis appropriately modelled caesarean sections as binary, and its results, presented on the odds ratio scale, should be considered the definitive estimates of intervention impact [6]. (3) The central limit theorem, upon which valid inference of sample means is based, may not hold with small sample sizes [29]. Our dataset has 105,351 patients from 32 independent clusters with a median cluster size of 2644 patients (range: 638 to 9608). (4) Sample size calculations for the QUARISMA trial were not designed to test cost-effectiveness hypotheses. Methodological guidance suggests that economic evaluation should focus on characterizing differences between study group, rather than formal hypothesis testing [21]. This trial had a large sample size, and overall and stratified cost-effectiveness analyses were able to detect a difference between study groups at a significance level of 0.05, suggesting that the sample size was adequate. (5) Our analysis was based on a health system perspective. Patients and providers may have personal preferences for caesarean sections, positive or negative, not captured by this analysis. (6) The main trial analysis identified a possible random increase in complications in the control group. Some proportion of the cost savings conferred by the intervention might be attributable to random variation; this cannot be ascertained from the trial data. (7) Hospitals eligible for inclusion in this trial had a minimum annual delivery volume of 300; intervention performance in smaller hospitals is unknown, and this may affect the scope for achieving benefits at scale. Returns from the QUARISMA trial itself may range from $0.5 million to $5 million in the 1-year post-intervention period alone (Additional file 1: Table S10). (8) This study was done in a single jurisdiction; differences in health system organization and financing may limit transferability of results to other settings. A national budget impact analysis is being conducted to translate these results for all Canadian provinces. (9) The relatively short trial time horizon does not elucidate how the intervention impact may evolve over time.

A similar program could be beneficial in other regions with similar or higher caesarean section rates [6]. Cost-effectiveness results can be adapted to other jurisdictions by adjusting for local baseline caesarean rates, health system costs, and proportions of high- and low-risk patients.

## Conclusions

From a healthcare payer perspective, a multifaceted intervention involving audits and feedback resulted in a small reduction in caesarean deliveries and an important reduction in per-patient costs. These results were achieved without increasing neonatal and maternal morbidity and mortality.

In keeping with the theory of change for the QUARISMA intervention, which attempted to optimize medical practice by reducing *unnecessary* caesareans, our study found that women with low-risk pregnancies experienced statistically significant reductions in caesarean sections and costs, while changes for the high-risk subgroup were not significant. We also found evidence of improved quality along the continuum of care. Cost reductions were driven principally by lower rates of neonatal complications and corresponding lower use of resources within the intervention group. These changes are consistent with improvements in the quality of care in intervention group hospitals.

Findings from our study provide critical new evidence concerning a safe and possibly sustainable strategy to reduce unnecessary caesarean sections and shed new light on the potential for audit and feedback interventions to improve quality of care while controlling costs. Delivery of the intervention at wider scale accompanied by further research is required to assess whether the improvements in clinical practice driving reductions in caesarean sections and cost savings are sustainable over time.

## Additional file

Additional file 1: Supplementary material for a cluster-randomized trial to reduce caesarean delivery rates in Quebec: cost-effectiveness analysis. (PDF 716 kb)

## Abbreviations

CIHI: Canadian Institute for Health Information
CS: Caesarean section
ICER: Incremental cost-effectiveness ratio
NPD: National Physician Database
PCE: Patient Cost Estimator
QUARISMA: Quality of Care, Obstetrics Risk Management, and Mode of Delivery (trial)
