# Supplementary material for: A cluster-randomized trial to reduce caesarean delivery rates in Quebec: cost-effectiveness analysis
Source: BMC Med. 2017 May 22;15:96. doi: 10.1186/s12916-017-0859-8 (PMC5439122; doi:10.1186/s12916-017-0859-8)
Supplement: Supplementary file 1 — Supplementary material for a cluster-randomized trial to reduce caesarean delivery rates in Quebec: cost-effectiveness analysis. (PDF 716 kb) [file 12916_2017_859_MOESM1_ESM.pdf]

SUPPLEMENTARY APPENDIX FOR THE STUDY: A CLUSTER-RANDOMIZED TRIAL TO REDUCE CAESAREAN DELIVERY RATES IN QUEBEC: COST-EFFECTIVENESS ANALYSIS (JOHRI ET AL., 2017)

Table of contents

SECTION 1: QUARISMA Trial research group 3

SECTION 2: Expansion on statistical analysis methods..5

SECTION 3: Expansion on methods for resource use & costing 7

Figure S1: Incremental cost-effectiveness of the QUARISMA intervention versus routine care (sensitivity analysis using costs from the Province of Quebec Billing Manual for Medical Specialists[14]) 8

Figure S2: Incremental cost-effectiveness of the QUARISMA intervention versus routine care, by risk subgroups (sensitivity analysis using costs from the Province of Quebec Billing Manual for Medical Specialists[14]) 9

Table S1: Clinical categories used in constructing the total cost variable 10

Table S2: Clinical categories and medical procedure costs used in the main analysis[a]................12

Table S3: Physician fee estimates used in main and sensitivity analyses, by clinical category 16

Table S4: QUARISMA intervention costs [a, b] 19

Table S5. Baseline characteristics of hospitals and patients enrolled in the QUARISMA trial[a, b] 20

Table S6. Impact of the QUARISMA intervention on cesarean sections and total direct medical costs, overall and by patient risk subgroups (sensitivity analysis using costs from the Province of Quebec Billing Manual for Medical Specialists)..................................22

Table S7. Changes in per-patient costs due to the intervention, by clinical category[a,b]................24

Table S8: Intraclass correlation (ICC) for costs and effects[a] 26

Table S9: Individual-level correlation between costs and effects[1] 27

Table S10: Return on Investment 28

References 29

SECTION 1: QUARISMA Trial research group

QUARISMA Economic Evaluation Committee: Mira Johri, Edmond Ng, Clara Bermudez-Tamayo, Jeffrey S. Hoch, Thierry Ducruet, Nils Chaillet. *Economic Evaluation Analyses:* Edmond Ng, Mira Johri, Clara Bermudez-Tamayo.

QUARISMA Main Trial Analysis (Chaillet et al, 2015): Writing Committee: Nils Chaillet, Alexandre Dumont, Michal Abrahamowicz, Jean-Charles Pasquier, François Audibert, Patricia Monnier, Haim A. Abenhaim, Eric Dubé, Marylène Dugas, Rebecca Burne, William D. Fraser. *Steering Committee*: Nils Chaillet, Alexandre Dumont, William D. Fraser, Patricia Monnier, François Audibert, François Beaudoin, Michal Abrahamowicz. *Statistical Analyses*: Michal Abrahamowicz, Rebecca Burne. *Coordination and Data Management*: Eric Dubé, Abbas Kerim-Dikeni, Thierry Ducruet, Francine Beaulieu, Amel Zertal, Muriel Koucoï, Joannah Valma, Pamela Palma, Maggy Wassef. *Data Safety and Monitoring Board*: José Villar (Chair), Fernando Althabe, Ahmadou Alioum.

Participating institutions and staff
University of Sherbrooke (Sherbrooke, Canada): Nils Chaillet, William D. Fraser, Jean-Charles Pasquier, Eric Dubé; Institut de recherche pour le développement (Paris, France): Alexandre Dumont; University of Montreal (Montreal, Canada): François Beaudoin, François Audibert, Robert Gauthier, Johanne Dubé, Louise Duperron, Diane Francoeur, Line Leduc, Isabelle Amyot, Marie-Josée Bédard, Marie-Claude Lemieux, Sonia Gagnon, François Champagne and Mira Johri (Public Health), Gilles Bibeau and Hélène Vadeboncoeur (Arts and sciences); McGill University (Montréal, Canada): Michal Abrahamowicz, Rebecca Burne, Haim A. Abenhaim; Laval University (Québec, Canada): Emmanuel Bujold, Normand Brassard, Marylène Dugas; McMaster University (Hamilton, Canada): Jean-Eric Tarride; University of Ottawa (Ottawa, Canada): Jeremy Grimshaw; Society of Obstetricians and Gynecologists of Canada (Ottawa, Canada): André Lalonde, Vyta Senikas, Guy-Paul Gagné (MORE^{ob}); Association des Obstétriciens et Gynécologues du Québec (Montréal, Canada): Diane Francoeur, Corinne Leclercq, Isabelle Girard; Association des Omnipraticiens en Périnatalité du Québec (Montréal, Canada): Andrée Gagnon; Ordre des Sages-Femmes du Québec (Montréal, Canada): Marie-Eve St Laurent, Raymonde Gagnon; Ministry of Health and Social Services (Québec, Canada): Jeannine Auger, Isabelle Côté; Institut national d'excellence en santé et services sociaux (Montréal, Canada): Jean-Marie Moutquin, Michel Rossignol.

Participating hospitals in Québec
Centre Hospitalier de Granby: Lucie Larouche, Nathalie Beaumont, Marianne Blais, Mélanie Gélinas, Monique Poulin, Sylvie Tardif; Centre Hospitalier de Val d'Or: Manon Turbide, Élisabeth Picard, Lizane Chrétien, Lyne Godbout, Yves Bolduc, Pierre-Charles Gosselin, Charbel Salameh, Louise Cardinal; Centre Hospitalier Hôtel-Dieu d'Amos: Pascale Gaudet, Serge Lamontagne, Bertrand Lavoie, Gisèle Delisle, Lyse Landry, Céline Bourrassa, Denise Lanoix; Centre Hospitalier Hôtel-Dieu d'Alma: Yves Bolduc, Sylvie Boudrault, Lucie Rouleau; Centre Hospitalier Hôtel-Dieu de Lévis: Maryse Turcotte, Henri Chamberland, Jean-Pierre Gennaoui, Louise Martin; Centre Hospitalier Hôtel-Dieu de Roberval: Benoît Brassard, Martin Rioux, Suzanne Gagné, Gilles Guillemette, Odette de GrandMaison; Centre Hospitalier Hôtel-Dieu de Sorel: Gérald Désaulniers, Jacques Claveau, Abdelmoula Khaled, Marion Laforce, Johanne Plammondon; Centre Hospitalier Hôtel-Dieu de St-Jérôme: Annick Hamel, Yannik Vézina, Anne Gosselin, Lynda Couture, Anne Piltan, Louis-André Lacasse, Emmanuelle Dubois, Karina Daigle; Centre Hospitalier Mont-Laurier: Marcel Cadotte, Chantal Aubry, Ludivine Dugas; Centre Hospitalier Pierre-Boucher: Michel Laurence, Bernard Couturier; Centre Hospitalier Pierre-Le Gardeur: Gerry Bédard, Guy Girard, Suzanne Limoge; Centre Hospitalier Régional Baie-Comeau: Roger Dubé, Suzanne Roberge, Mélanie Millaire, Martine Lévesque, Hélène Dangeault, Normande Lessard; Centre Hospitalier Régional de La Mauricie: Eric Kerkerian, Faouzi Zaidan, Diane Racine, Lisette Richard; Centre Hospitalier Régional de Lanaudière: Claude Gauthier, Valérie Hétu, Karine-Émmanuelle Boisvert, Isabelle Lamoureux, Chantal Gascon, Hélène Ammelin; Centre Hospitalier Régional de Rimouski: Stéphanie Lévesque, Isabelle Poirier, Kathleen Brisson, Janique Plourde, Robert Daignault, Roger Bouchard, Anne Groleau; Centre Hospitalier Régional de Sept-Îles: Pierre Gauthier, Isabelle Bossé, Michelle Martel, Hélène Robichaud, Dolorès Roy; Centre Hospitalier Régional de Trois-Rivières: Véronique Bussière, Natacha Malmedy, Dominique Giguère, Mélanie Thibault, Yves Lamirande, Michel Patry, Louise Gilbert; Centre Hospitalier Rouyn-Noranda: Annie Léger, Pierre Bouthiette; Centre Hospitalier Saint-Eustache: Karine Boily, Nathalie Ouellet, Isabelle Giguère, Edith Rochon, Majorie Nicole, Abdelghani El-Fakir; Centre Hospitalier St-Mary's: Bruce Brown, Robert Hemmings, Isabelle Girard; Centre Hospitalier Universitaire de Laval: Renée Grondin, Normand Brassard, Emmanuel Bujold, France Gagnon, Caroline Gilbert; Centre Hospitalier Universitaire

de Montréal: Marie-Josée Bédard, Yves Korcaz, Marie Claude Matte, Johanne Steben, Louise Valiquette, Tanya Lavades, Charles Bellavance, Serge Bélisle, Martine Campeau; Centre Hospitalier Universitaire de Sainte-Justine: Robert Gauthier, François Beaudoin, Louise Duperron, Catherine Taillefer, Christiane Grondin, Isabelle Amyot, Diane Francoeur, Line Leduc; Centre Hospitalier Universitaire de Sherbrooke: Jacques Poisson, Jean-Marie Moutquin, Jean-Charles Pasquier, Maryse Bertiaume; Hôpital Brome-Missisquoi-Perkins: Christine Cadrin, Christiane Charest, Geneviève Adam, Louise Galipeau, Marie-Claude Boudreau, Cynthia Carrier, Louise Lapointe, Christian Leger, Normand Chagnon, Andrée Guillault; Hôpital Cité de la Santé de Laval: Alain Goudreau, Dominique Tremblay, Jean-François Lanctôt, Maxime Lacerte, Gilles St-Jean, Diane Bertrand, Judy Blumenthal; Hôpital de Chicoutimi: Bernard Parent, Fabien Simard, Patricia Fisch; Hôpital de Saint-Georges: Marie-Claude Bélanger, Jean Grégoire; Hôpital Général du Lakeshore: Sylvie Douyon, Gilles Mercier, Ruth Bressnen; Hôpital Honoré-Mercier: Normand Kingsley, Lysanne Proulx, Jean-Guy Bibeau; Hôpital Maisonneuve-Rosemont: Ewa Sidorowicz, Anne Di Zazzo, Julie Guimond, Martine Bertrand, Sophia Aouinati, Gabriela Ponce; Hôpital Sainte-Croix: Luc Gilbert, Marie-Claude Philibert, Caroline Gadouri.

SECTION 2: Expansion on statistical analysis methods

QUARISMA is a cluster-randomized trial of a group-level intervention, where randomization was done at the level of the cluster (hospital) and outcomes recorded for individual patients. The trial compared two random cross-sections (baseline data on cesarean sections were collected during the 1 year pre-intervention period, and the primary outcome was the cesarean section rate assessed in the 1-year post-intervention period). Appropriate methods for cost-effectiveness analysis of cluster-randomized trials must address the potential correlation between costs and outcomes for individual patients, and correlations within clusters.[1,2]

The main analysis used a bivariate linear mixed (multilevel) model (BMLM) to estimate regression coefficients and permit calculation of incremental costs and benefits. This choice was motivated by recent work that has established that BMLMs are appropriate for cost-effectiveness analyses of data from cluster-randomized trials, providing unbiased parameter estimates and good confidence interval coverage (close to nominal level), over a range of simulated data scenarios including skewed costs and few clusters.[2] Cost data tends to be right-skewed and this was also true in our trial dataset; however, transformation and back transformation of cost data often do not provide satisfactory estimates and BMLMs are shown to be robust against skewness in costs as well as a range of other challenging data scenarios.[3]

In our BMLMs, correlations between patient-level costs and health outcomes as well as that between hospital-level mean costs and outcomes were modeled explicitly while recognizing clustering of the data within hospital centers through cluster-level random effects. The BMLMs were fitted first by restricted unbiased iterative generalized least-squares (RIGLS) estimation in MLwiN (version 2.35)[4]. Random effects at cluster as well as individual levels were assumed to follow bivariate normal distributions. We invoked MLwiN from Stata using the command, *runmlwin*, for reproducibility and ease of reporting.[5]

In the subsequent runs for ascertaining the uncertainty around the joint distribution of incremental costs and effects, Bayesian Markov chain Monte Carlo estimation (MCMC) was used to generate the joint posterior distribution of the estimands. Initial parameter values are required in MCMC estimation, the RIGLS estimates derived previously were used as initial values. Default 'diffuse' priors in MLwiN were used throughout. The default priors are flat or diffuse for all parameters. In particular, fixed parameters, β, were assumed to follow a Uniform distribution with $p(\beta) \propto 1$. Functionally this is the same as a Normal prior with an extremely large variance. For scalar variances, $p\left(\frac{1}{\sigma^2}\right) \sim \Gamma(\varepsilon, \varepsilon)$ where $\Gamma(\varepsilon, \varepsilon)$ is a Gamma distribution with shape and scale parameters both equal to ε. This is equivalent to a Uniform prior for log(σ^2) when ε takes on a very small value. For variance matrices, $p(\Omega^{-1}) \sim Wishart_p\left(p, p, \widehat{\Omega}\right)$ where p is the number of rows in the variance matrix and $\widehat{\Omega}$ is the initial value of Ω which is derived from RIGLS estimation in our analysis.

To construct the models, we first specify separate model equations for costs and effects for each person. The dependent variable "total costs" was constructed by summing resource use events for each patient (extracted from patient charts), multiplied by unit costs for each resource use category (identified through official Canadian government data sources for the province of Quebec[6,7]). The dependent variable "cesarean section" was taken from patient chart data. For graphical purposes and interpretation of the ICER, the outcome was rescaled such that a reduction in cesarean sections would indicate a more effective program.

We used BMLMs to calculate the adjusted differences-in-differences estimator (with 95%CI) of total costs and cesarean sections to obtain the change in total costs conferred by the intervention and the change in cesarean deliveries conferred by the intervention. The bivariate model equations are:

Crude Model

$$c_{ij} = \beta_0^c + \beta_1^c TX_j + \beta_2^c Time_{ij} + \beta_3^c TX_j \times Time_{ij} + u_j^c + \varepsilon_{ij}^c \qquad \begin{pmatrix} \varepsilon_{ij}^c \\ \varepsilon_{ij}^e \end{pmatrix} \sim BVN\left[\begin{pmatrix} 0 \\ 0 \end{pmatrix}, \begin{pmatrix} \sigma_c^2 & \rho\sigma_c\ \sigma_e \\ & \sigma_e^2 \end{pmatrix}\right]$$

$$e_{ij} = \beta_0^e + \beta_1^e TX_j + \beta_2^e Time_{ij} + \beta_3^e TX_j \times Time_{ij} + u_j^e + \varepsilon_{ij}^e$$

$$\begin{pmatrix} u_j^c \\ u_j^e \end{pmatrix} \sim BVN\left[\begin{pmatrix} 0 \\ 0 \end{pmatrix}, \begin{pmatrix} \tau_c^2 & \psi\tau_c\,\tau_e \\ & \tau_e^2 \end{pmatrix}\right]$$

where $i = 1,\ldots,n_j$ patients, $j = 1,\ldots,J$ clusters, u_j^c, u_j^e are the level 2 random effects for cluster j, and $\varepsilon_{ij}^c, \varepsilon_{ij}^e$ are the level 1 random effects for patient i in cluster j for cost and effect, respectively. τ_c^2 , τ_e^2 and ψ are the level 2 variances for costs and effects, and correlation between the level 2 random effects, u_j^c and u_j^e. σ_c^2 , σ_e^2 and ρ are the variances for costs and effects, and correlation at level 1. BVN stands for bivariate normal distribution.

Adjusted Model

$$c_{ij} = \beta_0^c + \beta_1^c TX_j + \beta_2^c Time_{ij} + \beta_3^c TX_j \times Time_{ij} + \beta_4^c X_{4ij} + \cdots + \beta_p^c X_{5ij} + u_j^c + \varepsilon_{ij}^c$$

$$e_{ij} = \beta_0^e + \beta_1^e TX_j + \beta_2^e Time_{ij} + \beta_3^e TX_j \times Time_{ij} + \beta_4^e X_{4ij} + \cdots + \beta_p^e X_{5ij} + u_j^e + \varepsilon_{ij}^e$$

$$\begin{pmatrix} \varepsilon_{ij}^c \\ \varepsilon_{ij}^e \end{pmatrix} \sim BVN\left[\begin{pmatrix} 0 \\ 0 \end{pmatrix}, \begin{pmatrix} \sigma_c^2 & \rho\sigma_c\;\sigma_e \\ & \sigma_e^2 \end{pmatrix}\right]$$

$$\begin{pmatrix} u_j^c \\ u_j^e \end{pmatrix} \sim BVN\left[\begin{pmatrix} 0 \\ 0 \end{pmatrix}, \begin{pmatrix} \tau_c^2 & \psi\tau_c\,\tau_e \\ & \tau_e^2 \end{pmatrix}\right]$$

The two outcomes (c_{ij} costs and e_{ij} cesarean sections) are modeled jointly. For the crude model, TX_j represents the intervention for cluster j (1 for intervention; 0 for usual care), $Time_{ij}$ designates the study period for patient i in cluster j (0 for baseline; 1 for post-intervention or end line), and the coefficient associated with the interaction term $TX * Time_{ij}$ (β_3^c for costs and β_3^e for effects) is the differences-in-differences estimator of incremental change in the outcome due to the intervention. The adjusted model included all terms in the crude model and X_{4ij}^r to X_{pij}^r, where r = c and e, and X_p is the last covariate included in the model. β_4^r to β_p^r ,where r= c and e, are the regression coefficients of corresponding covariates for costs and cesarean sections respectively. We used the same set of covariates for adjustment used in the main trial (risk of pregnancy (low or high), parity (number of previous live births), current smoking (yes or no), birth weight (in grams), and hospital type (academic, community, regional) to estimate both costs and effects.

To gain insight into the drivers of overall cost savings and shed light on QUARISMA's theory of change, we conducted an analysis to describe the evolution in costs for key cost subcategories. The analysis was empirically motivated. We divided the total cost variable into three mutually exclusive and collectively exhaustive subgroups: "costs associated with delivery mode (cesarean or vaginal)", "costs associated with management of maternal complications", and "costs associated with management of neonatal complications", which became the dependent variables for our regression analyses. For each of these three cost subgroups, analyses explored whether the change in costs in the intervention group from baseline to post-intervention differed from the change in costs in the control group from baseline to post-intervention. We computed crude and adjusted models using the same BMLMs as for the main analysis. Crude and adjusted models included four indicator variables describing mean costs for control and intervention groups at baseline and at post-intervention, and were otherwise defined as for the main analysis. We used Stata's *lincom* command to test the null hypothesis that the linear combination representing the difference in the rate changes [(control group at baseline – control group at post-intervention) – (intervention group at baseline – intervention group at post-intervention)] is equal to zero. An additional analysis used the same procedure to compare two mutually exclusive and collectively exhaustive subgroups comprising the neonatal complications category: "cardiopulmonary morbidity (cardiopulmonary resuscitation)" versus "no cardiopulmonary morbidity."

SECTION 3: Expansion on methods for resource use & costing

The economic evaluation considered resource use and costs associated with key clinical events (mode of delivery, maternal complications, and neonatal complications), as categorized in the QUARISMA main trial (Chaillet et al., 2015; Appendix Section 3: Composite risks of morbidity).[8] Table S1 presents the clinical categories established for the QUARISMA trial, and shows which categories were used to construct the total cost variable. Due to data limitations, we excluded three conditions, each with a frequency of < 0.5%.

Medical procedure costs for the main analysis were calculated as the sum of hospital costs from the CIHI Patient Cost Estimator (PCE)[9] and physician costs from the National Physician Database (NPD)[6], see (Table S2). Medical procedure costs for the sensitivity analysis were calculated as the sum of hospital costs from the CIHI Patient Cost Estimator[9] (Table S2) and physician costs from the Medical Specialists of Quebec Fee-for-Service Billing Manual (MSQ) [8], see (Table S3). Medical procedure costs were summed to generate total costs. We used PCE and NPD costs for the jurisdiction of Quebec.[10] MSQ costs are specific to the Province of Quebec.

To quantify hospital costs, we used individual patient data to refine information on clinical events to match the finest available case-mix codes and groupings in the PCE.[9] For example, cesarean section costs were modulated by patient age (adult or pediatric), use of induction, and presence of a uterine scar. Vaginal birth costs were modulated by patient age (adult or pediatric), use of anesthetic, additional interventions (e.g. episiotomy), and assisted delivery (e.g. forceps, vacuum extractor). Individual patients were then assigned the PCE[9] medical procedure costs corresponding to these refined case-mix groupings.

To quantify physician costs, we developed schema for typical clinical pathways and associated physician activities and billing practices. Physician contributions were assessed separately for NPD costs and MSQ costs; Table S3 presents a comparison. Schema were established in consultation with experts from the QUARISMA research team. Experts included Quebec physicians specializing in obstetrics and gynecology, pediatrics, neonatology, and family medicine.

Three maternal complications (postpartum hospital stay ≥ days; admission to ICU; readmission to hospital after postpartum discharge) were not available in the CIHI Patient Cost Estimator. For these procedures, we calculated costs by multiplying per diem values by patient length of stay. We made a data request to the Canadian Institutes of Health Information for 2011-2012 per diem costs for acute care hospitals from the neighboring province of Ontario, as values for Quebec were not available.[11] Data on length of stay were calculated from individual patient data from the QUARISMA trial, and in once instance (admission to ICU) from the scientific literature.[12]

CIHI Patient Cost Estimator and MSQ costs were in 2013 Canadian dollars. We converted 2011-2012 NPD costs to 2013 dollars, by applying the Bank of Canada's overall Consumer Price Index. The CPI for June 2011 was 119.8 and for June 2013 123.0, implying a 2.67% change.[13]

Figure S1: Incremental cost-effectiveness of the QUARISMA intervention versus routine care (sensitivity analysis using costs from the Province of Quebec Billing Manual for Medical Specialists[14])

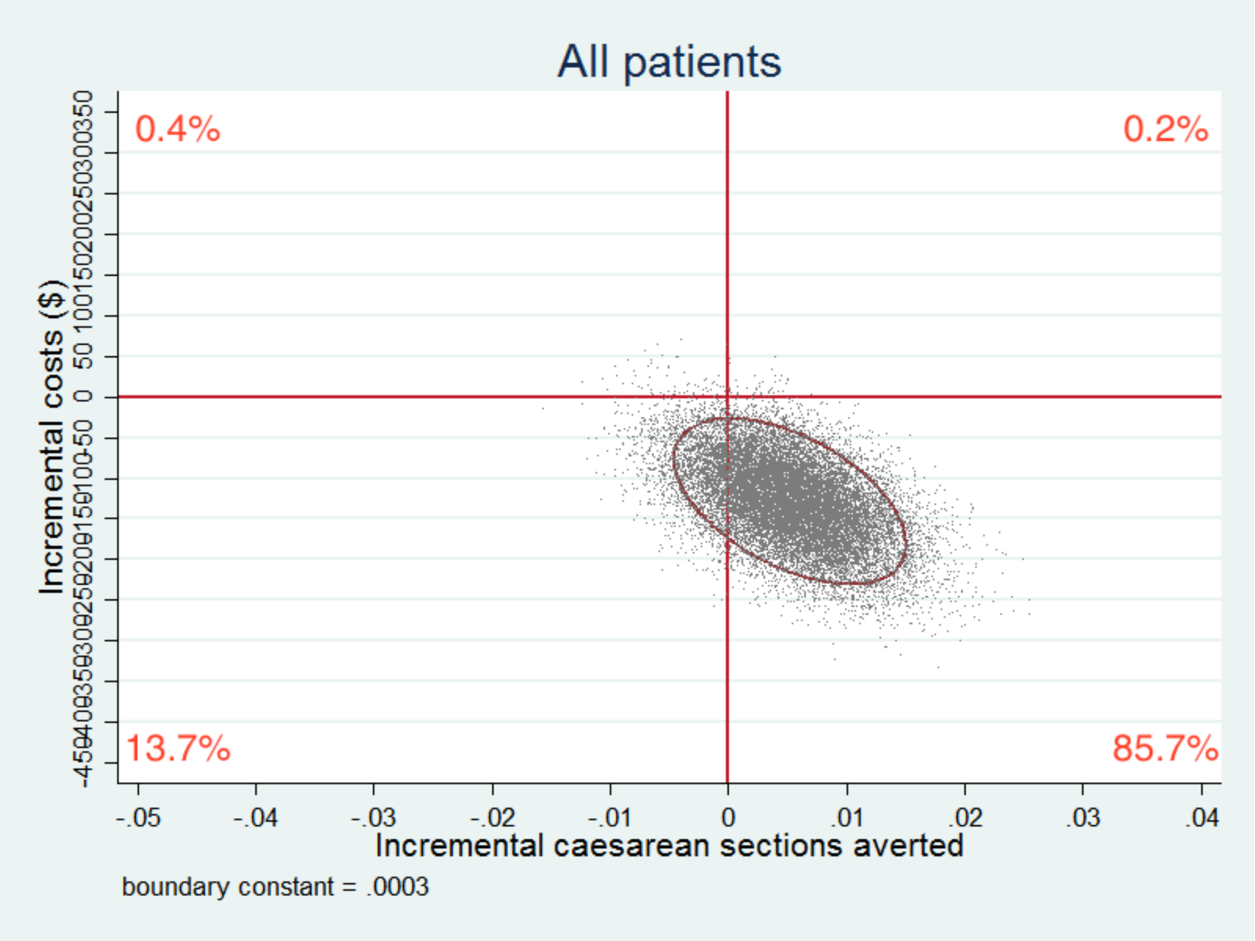

Figure S1. Cost-effectiveness (CE) plane for the covariate adjusted cost-effectiveness analysis of the QUARISMA intervention versus routine care. Incremental cost-effectiveness results were based on 1000 bootstrap replicates including all trial participants (N=105,351). Uncertainty on the CE plane is represented by an ellipse containing 95% of the joint density of cost and effect differences. The center of the ellipse represents the point estimate of incremental effects and costs, i.e. a per-patient reduction of 0.005 cesarean sections and $128 saved. Percentages represent the distribution of points by quadrant.

Figure S2: Incremental cost-effectiveness of the QUARISMA intervention versus routine care, by risk subgroups (sensitivity analysis using costs from the Province of Quebec Billing Manual for Medical Specialists[14])

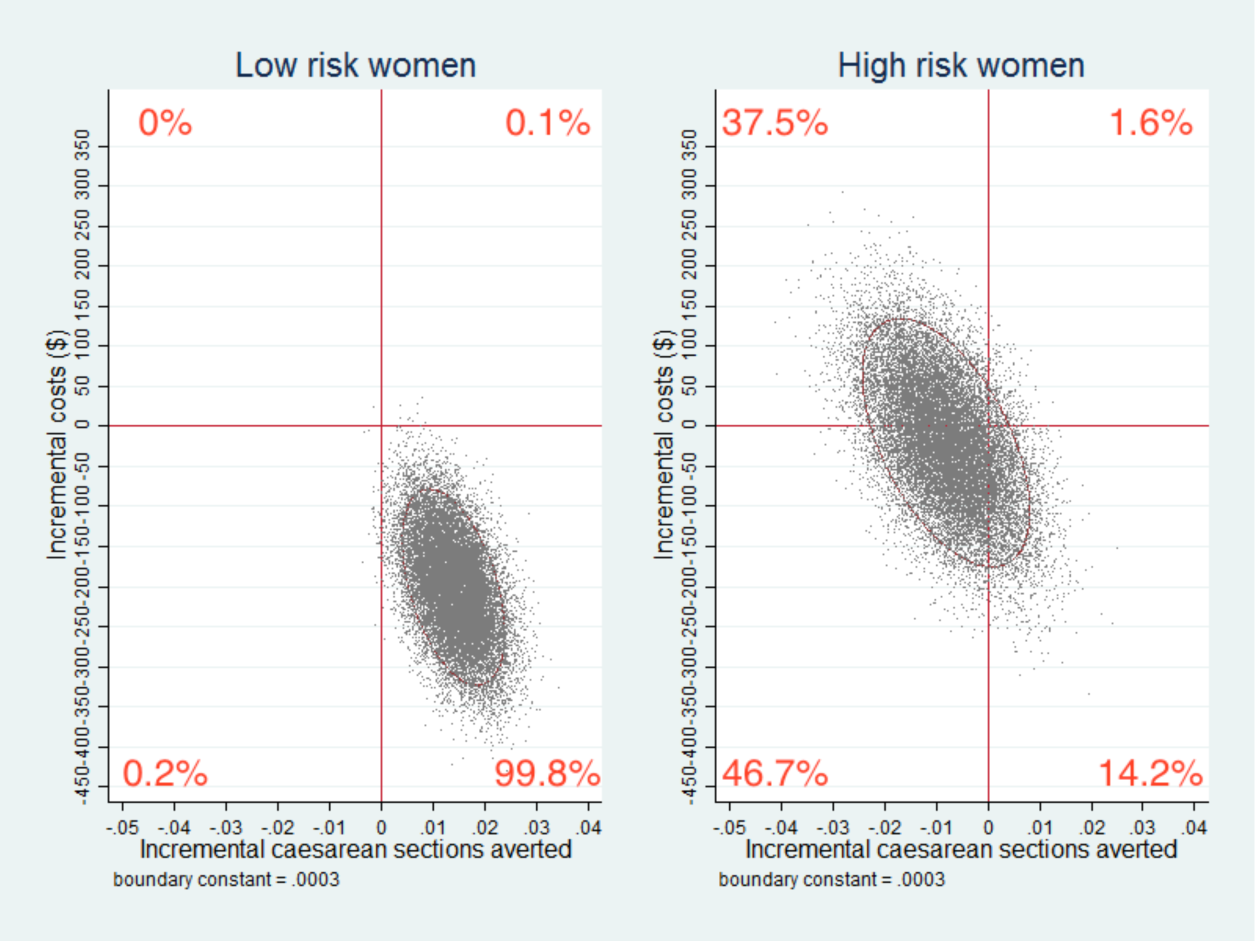

Figure S2. Cost-effectiveness (CE) planes for the covariate adjusted cost-effectiveness analysis of the QUARISMA intervention versus routine care, by patient risk subgroups. Incremental cost-effectiveness results based on 1000 bootstrap replicates estimated separately for low risk (N=49,281) and high-risk (N=56,070) trial participants. Uncertainty on the CE plane is represented by an ellipse containing 95% of the joint density of cost and effect differences. The center of the ellipse represents the point estimate of incremental effects and costs, i.e. a per-patient reduction of 0.014 cesarean sections and $201 saved for the low-risk subgroup, and a per-patient increase of 0.008 cesarean sections and $19 saved for the high-risk subgroup. Percentages represent the distribution of points by quadrant; figures may not sum to 100 due to rounding.

Table S1: Clinical categories used in constructing the total cost variable

Clinical event category[a]	Frequency n	(%)[b]	Included in total cost variable ?
**Mode of delivery**	**N = 105,351**		
Cesarean	24,050	(22.83)	Yes
Vaginal	81,301	(77.17)	Yes
**Minor maternal morbidity**	**N = 105,351**		
Blood transfusion	868	(0.82)	Yes
Perineal tear (grade 3-4)	5,490	(5.21)	Yes
Puerperal infection / sepsis	7,981	(7.58)	Yes
Gastrointestinal complications[c]	296	(0.28)	No
Analgesia complications[c]	380	(0.36)	No
Postpartum hospital stay >=7 days	676	(0.64)	Yes
Admission to ICU	489	(0.46)	Yes
Readmission to hospital after postpartum discharge	1,348	(1.28)	Yes
**Major maternal morbidity**	**N = 105,351**		
Maternal death (per 100 000)	6[d]	--	Yes
Hysterectomy	62	(0.06)	Yes
Symptomatic uterine rupture	21	(0.02)	Yes
Thromboembolic disease	241	(0.23)	Yes
Internal organs injuries	298	(0.28)	Yes
**Minor neonatal morbidity**	**N=107,043**		
Cardiopulmonary morbidity	11,357	(10.61)	Yes
APGAR score (4 to 7)	1,653	(1.54)	Yes
Moderate acidosis (7 to 7.1)	1,440	(1.35)	Yes
Minor trauma	1,078	(1.01)	Yes
Non-invasive mechanical ventilation	5,869	(5.48)	Yes
Blood transfusion	175	(0.16)	Yes
Neonatal infection/ sepsis	3,223	(3.01)	Yes
**Major neonatal morbidity**	**N=107,043**		
Intrapartum/ neonatal death	97	(0.09)	Yes
APGAR score (<4)	692	(0.65)	Yes
Major acidosis (PH<7)	321	(0.30)	Yes
Major trauma	977	(0.91)	Yes

Intraventricular hemorrhage	94	(0.09)	Yes
Seizure at less than 24 hours	235	(0.22)	Yes
Neurologic damage[c]	456	(0.43)	No
Invasive mechanical ventilation	1,396	(1.30)	Yes
Necrotising enterocolitis	125	(0.12)	Yes
Hypoxic-ischemic encephalopathy	35	(0.03)	Yes

[a] These are clinical event categories considered to trigger resource use

[b] This is the frequency of occurrence among the 105,351 QUARISMA trial participants. Due to multiple births, the total number of neonates among trial participants is 107,043

[c]Gastrointestinal complications, analgesia complications, and neonatal neurologic damage were not considered due to the rarity of these conditions and data limitations

[d] There were 5.7 maternal deaths per 100 000 patients

Table S2: Clinical categories and medical procedure costs used in the main analysis[a]

Case mix group code & description[b]	Hospital cost ($)[c]	Physician fee ($)[d]	Total cost ($)[e]
**Procedures for the mother**			
**Delivery**			
577 Normal Newborn, Multiple/ CS Delivery (Newborn/ Neonate)	1,015		
Cesarean section (CS)		705	
558 Primary CS, with induction (Adult)	4,336		6,056
558 Primary CS, with induction (Pediatric)	4,925		6,645
559 Primary CS, no induction (Adult)	3,486		5,206
559 Primary CS, no induction (Pediatric)	3,564		5,284
560 CS with uterine scar, no induction (Adult)	2,841		4,561
560 CS with uterine scar, no induction (Pediatric)	2,895		4,615
561 CS with uterine scar, and induction (Adult & Pediatric)	3,968		5,688
Vaginal birth (VB)		776	
562 VB with Anesthetic and Non-Major Obstetric/Gynecologic Intervention (Pediatric)	2,951		4,742
562 VB with Anesthetic and Non-Major Obstetric/Gynecologic Intervention (Adult)	2,533		4,324
563 VB with Anesthetic without Non-Major Obstetric/Gynecologic Intervention (Adult)	2,107		3,898
563 VB with Anesthetic without Non-Major Obstetric/Gynecologic Intervention (Pediatric)	2,436		4,227
564 VB without Anesthetic with Non-Major Obstetric/Gynecologic Intervention (Adult)	2,144		3,935
564 VB without Anesthetic with Non-Major Obstetric/Gynecologic Intervention (Pediatric)	1,928		3,719
565 VB without Anesthetic without Non-Major Obstetric/Gynecologic Intervention (Adult)	1,670		3,461
565 VB without Anesthetic without Non-Major Obstetric/Gynecologic Intervention (Pediatric)	1,481		3,272

Case mix group code & description[b]	Hospital cost ($)[c]	Physician fee ($)[d]	Total cost ($)[e]
**Other maternal procedures[f]**			
Blood transfusion [g]	246	142	388
Perineal Tear Grades 3 & 4: 511 Vulva/Perineum Intervention (Adult)	1,516	381	1,897
Perineal Tear Grades 3 & 4: 511 Vulva/Perineum Intervention (Pediatric)	1,663	381	2,044
Puerperal Infection / Sepsis (Adult) [h]	4,945	254	5,199
Puerperal Infection / Sepsis (Pediatric) [h]	5,576	254	5,830
653 Septicemia due to StaphylococcusAureus/Pseudomonas/Enterococcus (Adult)	9,115		
653 Septicemia due to StaphylococcusAureus/Pseudomonas/Enterococcus (Pediatric)	8,269		
654 Other/Unspecified Septicemia (Adult)	8,109		
654 Other/Unspecified Septicemia (Pediatric)	11,560		
660 Other Infectious/Parasitic Disease (Adult)	4,374		
660 Other Infectious/Parasitic Disease (Pediatric)	4,685		
662 Fever (Adult)	2,990		
662 Fever (Pediatric)	3,909		
Hysterectomy: 502 Hysterectomy with Non Malignant Diagnosis (Adult)	3,827	1,100	**4,927**
Hysterectomy: 502 Hysterectomy with Non Malignant Diagnosis (Pediatric)	5,541	1,100	**6,641**
Symptomatic Uterine Rupture[i]: 507 Repair/Brachytherapy/Other Intervention on Female Reproductive System except Tube/Ovary (Adult)	2,489	1,152	**3,641**
Symptomatic Uterine Rupture[i] : 507 Repair/Brachytherapy/Other Intervention on Female Reproductive System except Tube/Ovary (Pediatric)	2,725	1,152	**3,877**
Thromboembolic Disease (Adult) [j]	4,716	576	**5,292**
Thromboembolic Disease (Pediatric) [j]	5,873	576	**6,449**
211 Deep Vein Thrombophlebitis (Adult)	4,089		
211 Deep Vein Thrombophlebitis (Pediatric)	3,599		
210 Embolism/Thrombosis except Deep Vein Thrombophlebitis (Adult)	5,342		
210 Embolism/Thrombosis except Deep Vein Thrombophlebitis (Pediatric)	8,146		
Internal Organs Injuries[k](Adult)	5,307	812	**6,119**
Internal Organs Injuries[k] (Pediatric)	4,248	812	**5,060**
454 Major Intervention on Upper Urinary Tract (Adult)	7,883		
454 Major Intervention on Upper Urinary Tract (Pediatric)	6,993		
455 Non-Major Intervention on Upper Urinary Tract (Adult)	4,732		
455 Non-Major Intervention on Upper Urinary Tract (Pediatric)	6,759		
457 Major Intervention on Lower Urinary Tract (Adult)	6,098		
457 Major Intervention on Lower Urinary Tract (Pediatric)	3,986		
458 Non-Major Intervention on Lower Urinary Tract,	2,784		

Case mix group code & description[b]	Hospital cost ($)[c]	Physician fee ($)[d]	Total cost ($)[e]
Unplanned (Adult)			
458 Non-Major Intervention on Lower Urinary Tract, Unplanned (Pediatric)	3,257		
467 Other Intervention with Urinary System Diagnosis (Adult)	8,300		
467 Other Intervention with Urinary System Diagnosis (Pediatric)	4,343		
506 Bladder Fixation	2,884		
230 Repair/Fixation & Other Moderate Intervention on Lower Gastrointestinal Tract (Adult)	4,468		
230 Repair/Fixation & Other Moderate Intervention on Lower Gastrointestinal Tract (Pediatric)	5,254		
**Maternal complications estimated using per diem costs[l]**	**Per diem[m]**	**Length of stay**	
Postpartum hospital stay > 7 days	947	Variable (based on trial data)	
Admission to Intensive Care Unit (ICU)	3,534	3.5 days[n]	
Readmission to hospital after postpartum discharge	947	Variable (based on trial data)	
**Procedures for the neonate**			
Transient tachypnea: 589 Newborn/Neonate, Major Respiratory Complication	2,657	4,381	7,038
Respiratory insufficiency or distress syndrome: 589 Newborn/Neonate, Major Respiratory Complication	2,657	8,066	10,723
Cardiopulmonary resuscitation: 590 Newborn/Neonate, Aspiration Syndrome/Fetal Asphyxia	2,495	119	2,614
Neonatal infection/Sepsis: 600 Newborn/Neonate 2500+ grams, Other Moderate Problem	4,770	1091	5,861
Subdural hematoma: 599 Newborn/Neonate, other major problem	6,604	664	7,268
Intracerebral or intraventricular hemorrhage: 599 Newborn/Neonate, other major problem	6,604	664	7,268
Spinal-cord injury: 599 Newborn/Neonate, other major problem	6,604	1,091	7,695
Brachial plexus injury/palsy and peripheral-nerve injury: 599 Newborn/Neonate other major problem	6,604	1,091	7,695
Basal skull fracture: 600 Newborn/Neonate 2500+ grams, Other Moderate Problem	4,770	699	5,469
Necrotizing enterocolitis : 599 Newborn/Neonate, other major problem	6,604	1,091	7,695
Hypoxic-ischemic encephalopathy : 599 Newborn/Neonate, other major problem	6,604	1,091	7,695
Invasive mechanical ventilation: 556 Diagnosis With Non-Major Obstetric/Gynecologic Intervention	2,319	--	2,319

[a] All costs given in 2013 Canadian dollars = (0.94 USD)[15]

[b] Unless otherwise stated, these are Canadian Institutes for Health Information (CIHI) categories.[9] Adult (18-59 years); Pediatric (8-17 years); Newborn/ Neonate (0-7 days).[9]

[c] Source: CIHI *Patient Cost Estimator 2013* unit cost estimates for the Province of Quebec.[9]

[d] Source: CIHI *National Physician Database 2011-2012* physician fees for the Province of Quebec.[6] Values were converted to 2013 Canadian dollars by applying the Bank of Canada's Consumer Price Index See http://www.bankofcanada.ca/rates/related/inflation-calculator/

[e] For the categories cesarean sections and vaginal births, total costs associated with a specific procedure are the sum of hospital costs, physician fees, and delivery costs (code 577). For other maternal and neonatal procedures, total costs are the sum of hospital costs and physician fees.

[f] Maternal death costs were distributed among the other categories

[g] Transfusion costs from the Centre for Transfusion Research at the University of Ottawa.

[h] Based on expert guidance, this is a weighted average of the unit costs for the codes (653*0.1 + 654*0.1 +660*0.6 + 662*0.2)

[i] Can be managed through surgical repair or hysterectomy. For the QUARISMA trial, management through hysterectomy was classified as hysterectomy.

[j] Trial data did not distinguish between pulmonary embolism and deep vein thrombosis. Based on expert guidance, we took an average of the unit costs for the codes (211*0.5 + 210*0.5).

[k] "Internal organs injuries" included extension of the uterine incision with laceration of one or both uterine arteries, or laceration of the bladder, ureter, or bowel. Trial data did not permit us to distinguish between sub-categories. Based on expert guidance, we took an average of the unit costs for the applicable codes ((454 + 455 + 457 + 458 + 467 + 506 + 230)/7)

[l] For these complications, we calculated individual patient costs by multiplying a per Diem rate by the length of stay.

[m] For Fiscal year 2011-2012, Ontario's per Diem rates for acute care hospitals were on average $1109. This is a weighted average of the component costs $3534 (ICU) and $947 (ward (non-ICU)).[11]

[n] Trial data did not record the length of an ICU stay. This is the average length of an ICU stay in the USA from a retrospective cohort study including 137 ICUs over the period 2002-2004.[12]

Table S3: Physician fee estimates used in main and sensitivity analyses, by clinical category

Procedures for the mother

Delivery

Procedure[a]	National Physician Database[b]	Medical Specialists of Quebec Billing Manual[c]
**Cesarean section**	$ 687.0	
Primary, with induction, <18 yrs		06912 Cesarean section with or without sterilization: $450<br>06946 Induction $100<br>70000 Analgesia $40
Primary, with induction, adult		06912 Cesarean section with or without sterilization: $450<br>06946 Induction $100<br>70000 Analgesia $40
Primary, no induction, <18 yrs		06912 Cesarean section with or without sterilization: $450<br>70000 Analgesia $40
Primary, no induction, adult		06912 Cesarean section with or without sterilization: $450<br>70000 Analgesia $40
with uterine scar, no induction, <18 yrs		06912 Cesarean section with or without sterilization: $450<br>Induction $100<br>70000 Analgesia $40
with uterine scar, no induction, adult		06912 Cesarean section with or without sterilization: $450<br>Analgesia $40
with uterine scar, and induction		06912 Cesarean section with or without sterilization: $450<br>Induction $100<br>70000 Analgesia $40
**Vaginal birth**	$ 756.1	
with anesthetic, non-major obst./gyn Intervention, <18 yrs		06903 Delivery: $400<br>Supplement: $100<br>70000 Analgesia $40
with anesthetic, non-major obst./gyn Intervention adult		06903 Delivery: $400<br>06946 Supplement: $100<br>70000 Analgesia $40
with anesthetic without non-major obst./gyn Intervention <18 yrs		06903 Delivery: $400<br>70000 Analgesia $40

Procedure[a]	National Physician Database[b]	Medical Specialists of Quebec Billing Manual[c]
with anesthetic without Non-Major obst./gyn Intervention adult		06903 Delivery: $400<br>70000 Analgesia $40
without anesthetic with Non-Major obst./gyn Intervention <18 yrs		06903 Delivery: $400<br>06946 Supplement: $100
without anesthetic with Non-Major obst./gyn Intervention adult		06903 Delivery: $400<br>06946 Supplement: $100
without anesthetic without non-major obst./gyn Intervention <18 yrs		06903 Delivery: $400
without anesthetic without Non-Major obst./gyn Intervention adult		06903Delivery: $400
Assisted, with anesthetic, non-major obst./gyn intervention <18 yrs		06903 Delivery: $400<br>06925 Supplement: $100<br>70000 Analgesia $40
Assisted, with anesthetic, non-major obst./gyn intervention adult		06903 Delivery: $400<br>06925 Supplement: $100<br>06925 Supplement: $100<br>70000 Analgesia $40
Assisted, without anesthetic with Non-Major obst./gyn intervention <18 yrs		06903 Delivery: $400<br>06925 Supplement: $100<br>70000 Analgesia $40
Assisted, without anesthetic with Non-Major obst./gyn intervention adult		06903 Delivery: $400<br>06925 Supplement: $100<br>70000 Analgesia $40
**Other maternal procedures**		
Blood Transfusion	Hospital Inpatient - Other (major assessment) : $86.34<br>Total Diagnostic/ Therapeutic services:<br>$51.81	No additional costs applied
Perineal Tear Grades 3 & 4	Hospital Inpatient - Other (major assessment) : $86.34<br>Surgical Assistance (nerve blocks / anesthesia) : $200.77<br>Other Minor Surgery : $84.31	06929 Obstetrical intrapartum care $425
Puerperal Infection/ Sepsis	Total Diagnostic/ Therapeutic services:<br>$51.81<br>2 Major Consultations : $195.60	06929 Obstetrical intrapartum care $425<br>AND<br>(06945 Supplement for complex cases $180 OR 09160 Initial visit and follow up consultation in microbiology / infectious diseases $175.60)

Procedure[a]	National Physician Database[b]	Medical Specialists of Quebec Billing Manual[c]
Hysterectomy	Surgical Assistance (nerve blocks / anesthesia) : $200.77<br>Hysterectomy: $538.23<br>Miscellaneous services $331.80	06099: Hysterectomy following a delivery complication or cesarean $1000
Symptomatic Uterine Rupture	Surgical Assistance (nerve blocks / anesthesia): $200.77<br>Miscellaneous services $331.80<br>Other Major Surgery : $589.75	06929 Obstetrical intrapartum care $425<br>06945 Supplement for complex cases $180
Thromboembolic Disease	Major Consultations: $97.80<br>ICU/ Resuscitation: $131.21<br>Miscellaneous services $331.80	06929 Obstetrical intrapartum care $425<br>06945 Supplement for complex cases $180
Internal Organs Injuries	Surgical Assistance (nerve blocks / anesthesia): $200.77<br>Other Major Surgery : $589.75	06929 Obstetrical intrapartum care $425<br>06945 Supplement for complex cases $180
**Procedures for the neonate**		
Transient tachypnea	$ 4267	03181 Transient tachypnea $1080.0
Respiratory insufficiency or distress syndrome	$ 7856	03181 Respiratory insufficiency or distress syndrome $1080.0
Cardiopulmonary resuscitation	$ 114.9	03171 Cardiopulmonary resuscitation $112.0
Subdural hematoma	$ 589.7<br>$ 57.6 ICU	07507 Subdural hematoma $ 560,0
Intracerebral or intraventricular hemorrhage	$ 589.7<br>$ 57.6 ICU	07507 Intracerebral or intraventricular hemorrhage $ 560,0
Spinal-cord injury	$1005.5<br>$ 57.6 ICU	02182 Spinal-cord injury $1 000,0
Basal skull fracture	$ 624<br>$ 57.6 ICU	02518 Basal skull fracture $ 600,0
Neonatal infection	$1005.5<br>$ 57.6 ICU	15254- 15257 Infection $1100,0

[a] These are Canadian Institutes for Health Information (CIHI) categories.[9]

[b] Source: CIHI *National Physician Database 2011-2012* physician fees for the Province of Quebec.[6] These are unadjusted 2011=2012 costs (directly as given in the source). The analysis used CPI-adjusted 2013 Canadian dollars as presented in Table S2.

[c] Source: Régie de l'assurance maladie du Québec. *Manuel de facturation: Rémunération à l'acte (médecins spécialistes)*. MAJ 85 ed2013.[14]

Table S4: QUARISMA intervention costs [a, b]

Activity	Cost per hospital ($)
**Pre-intervention phase**	
• Update of clinical practice guidelines	1,850
**Intervention phase**	
• Training and recertification of audit committee members and data collectors	12,406
• Facilitation by the Canadian Society of Obstetrics & Gynecology (instructors, monitors, educational outreach, and administrative and logistical support)	31,382
• Local audit committees (data collection, assessment and audit, formulation of recommendations, feedback to health professionals)	2,788 (± 2,360)
**Data collection and management**	
• Training of data collectors (nurses)	2,753
• Supervision, data collection, and quality control	34,376
• Data management	3,900
**Total**	**89,455 (± 2,360)**

[a] Values represent average costs per hospital. Plus-minus values given as mean ± standard deviation

[b] All costs given in 2013 Canadian dollars = (0.94 USD)[15]

Table S5. Baseline characteristics of hospitals and patients enrolled in the QUARISMA trial[a, b]

	Intervention		Control	
	Hospitals (N=16)	Patients (N = 24,388)	Hospital s (N=16)	Patients (N = 28,698)
**Hospital characteristics**				
Type of hospital				
Community (n = 4)	2	1325 (5.4)	2	803 (2.8)
Regional (n = 22)	11	16045 (65.8)	11	18684 (65.1)
Tertiary care (n = 6)	3	7018 (28.8)	3	9211 (32.1)
Academic status				
Yes	5	8977 (36.8)	4	16159 (56.3)
No	11	15411 (63.2)	12	12539 (43.7)
**Patient characteristics**				
Maternal age at delivery				
Mean age (years)		29.4 ± 5.1		29.8 ± 4.9
Range				
≤ 17 yr		255 (1.0)		122 (0.4)
18-34 yr		20,777 (85.2)		24370 (84.9)
≥ 35 yr		3,356 (13.8)		4206 (14.7)
Parity				
0		10,727 (44.0)		13,165 (45.9)*
1		8,893 (36.5)		10,607 (37.0)
≥ 2		4,768 (19.5)		4,926 (17.1)
Gestational age at delivery (weeks)				
< 37		2,069 (8.5)		1,982 (6.9)
37-41		22,269 (91.3)		26,675 (93.0)
≥ 42		50 (0.2)		41 (0.1)
Previous cesarean delivery		2,782 (11.4)		3,306 (11.5)
Cesarean deliveries				
Total		5,484 (22.5)		6,671 (23.2)
Elective		2,939 (12.1)		3,701 (12.9)
Intra-partum		2,545 (10.4)		2,970 (10.3)
High-risk pregnancy [c]		12,911 (52.9)		13,981 (48.7)
**Patient costs**[d]				
(mean ± SD, (IQR))				
Delivery (hospital costs)		4,232 ± 679 (3,252-6,627)		4,262 ± 696 (3,252-6,627)
Cesarean section		1,167 ± 2214 (0-6,627)		1,261±2281 (0-6,627)
VBAC		3,064±1,648 (0-4,722)		3,001 ±1689 (0-4,722)

SD – standard deviation; IQR – interquartile range; VBAC – vaginal birth after cesarean

[a] Hospital and patient characteristics reproduced from [8].

[b] Values given as number (%) unless otherwise stated. Percentages may not sum to 100 due to rounding. Plus-minus values are means ± SD. The P value patient characteristics was calculated by means of a univariate model with the use of generalized

estimating equations in which the structure for patient characteristics was exchangeable or independent. The P-value for cost data was calculated using the non-parametric Mann–Whitney U test. Using a threshold of $P<0.05$, there were no significant between-group differences at baseline except with regard to parity.

[c]A pregnancy was defined as "low risk" if the woman gave birth to a single baby in cephalic presentation, had not used assisted reproductive technology, was between 18 and 39 years of age, had a body-mass index before pregnancy between 17 and 29, had no prior cesarean delivery, no prior or current stillbirth, no transfer to another hospital during pregnancy, no other pathologic condition or complication during the current pregnancy or a prior pregnancy, and if the gestational age was between 37 and 42 weeks. A pregnancy was considered to be at risk if any of these conditions was not met.[8]

[d] All costs given in 2013 Canadian dollars = (0.94 USD)[15]

Table S6. Impact of the QUARISMA intervention on cesarean sections and total direct medical costs, overall and by patient risk subgroups (sensitivity analysis using costs from the Province of Quebec Billing Manual for Medical Specialists)

	Model 1: BMLM (Crude)				Model 2: BMLM (Adjusted)			
	**Coef. (ß)**	**Std. Err**	**95% CI**	**P-value**	**Coef. (ß)**	**Std. Err**	**95% CI**	**P-value**
**All participants**[c] (N=105,351 patients; 32 hospitals) [1]								
Effects (CS)	-0.009	0.005	(-0.019 to 0.002)	0.096	-0.005	0.005	(-0.015 to 0.004)	0.288
Costs ($)	-145	52	(-247 to -43)	0.005	-128	50	(-227 to -30)	0.011
**Low-risk subgroup**[d] (N=49,281 patients; 32 hospitals)								
Effects (CS)	-0.013	0.005	(-0.023 to -0.003)	0.013	-0.014	0.005	(-0.024 to -0.004)	0.005
Costs ($)	-183	63	(-306 to -60)	0.003	-201	61	(-321 to -80)	0.001
**High-risk subgroup**[d] (N=56,070 patients; 32 hospitals)								
Effects (CS)	0.009	0.008	(-0.007 to 0.024)	0.291	0.008	0.008	(-0.008 to 0.024)	0.307
Costs ($)	-22	78	(-174 to 131)	0.782	-19	77	(-170 to 132)	0.803

BMLM- Bivariate multilevel linear model; CS= cesarean section

[a] All costs given in 2013 Canadian dollars = (0.94 USD)[1]

[2] Total costs calculated using the Québec Medical Specialists Billing Manual (Régie de l'assurance maladie du Québec. *Manuel de facturation: Rémunération à l'acte (médecins*

[c] Adjusted models for all participants included the following covariates: parity, smoking, birth weight, hospital type, and women's risk level.

[d] Adjusted subgroup models included the following covariates: parity, smoking, birth weight, and hospital type.

Table S7. Changes in per-patient costs due to the intervention, by clinical category[a,b]

Cost Component	BMLM (Crude) Coef. (ß) ($)	Std.Err ($)	95% CI ($)	P-value	BMLM (Adjusted) Coef. (ß) ($)	Std.Err ($)	95% CI ($)	P-value[c]
**All participants (N=105,351 patients; 32 hospitals)**								
Delivery (cesarean or vaginal)[d]	-38.9	24.3	(-86.6 to 8.8)	0.11	-21.7	22.8	(-66.5 to 23.2)	0.34
Maternal complications[e]	18.7	30.7	(-41.5 to 79.0)	0.54	14.7	30.6	(-45.3 to 74.6)	0.63
Neonatal complications[f]	-125	25.2	(-174 to -75.4)	<0.001	-121	24.9	(-170 to -72.5)	<0.001
**Low-risk group (N=49,281 patients; 32 hospitals)**								
Delivery (cesarean or vaginal)[d]	-68.4	24.7	(-117 to -19.9)	0.006	-74.9	24.2	(-122 to -27.5)	-68.4
Maternal complications[e]	22.5	39.5	(-54.9 to 99.9)	0.57	14.8	39.1	(-61.9 to 91.5)	22.5
Neonatal complications[f]	-138	31.6	(-200 to -75.8)	<0.001	-141	31.5	(-202 to -78.9)	<0.001
**High-risk group (N=56,070 patients; 32 hospitals)**								
Delivery (cesarean or vaginal)[d]	48.5	37.2	(-24.3 to 121)	0.19	48.1	37.2	(-24.8 to 121)	0.20
Maternal complications[e]	15.3	46.2	(-75.3 to 106)	0.74	11.6	46.1	(-78.7 to 102)	0.80
Neonatal complications[f]	-84.9	38.1	(-160 to -10.3)	0.026	-78.4	37.3	(-152 to -5.3)	0.036

BMLM- Bivariate multilevel linear model; CI – Confidence Interval

[a] Model coefficients **(ß),** standard errors, and 95% confidence intervals present costs given in 2013 Canadian dollars = (0.94 USD)[1]

[b] Total costs calculated using the Québec Medical Specialists Billing Manual (Régie de l'assurance maladie du Québec. *Manuel de facturation: Rémunération à l'acte (médecins spécialistes).* MAJ 85 ed2013).[14]

[c] The p-value is the probability of observing a result at least as extreme as the z-statistic, for the null hypothesis that the difference in the rate changes in costs for the intervention group from baseline to post-intervention versus the control group from baseline to post-intervention does not differ from zero.

[d] Delivery costs include: cesarean delivery (primary or secondary, with or without induction, with or without uterine scar, pediatric or adult); vaginal delivery (with or without anesthetic, with or without intervention, assisted or unassisted, pediatric or adult).

[e] Maternal complication costs include: maternal death, hysterectomy, symptomatic uterine rupture, thromboembolic disease, internal organs injuries, perineal tear (grades 3-4), puerperal infection/ sepsis, postpartum hospital stay >=7 days, admission to ICU, readmission to hospital after postpartum discharge, blood transfusion.

[f] Neonatal complication costs include: intrapartum/ neonatal death, APGAR score (<4; 4 to 7), major and minor acidosis (PH<7; PH 7 to 7.1), major and minor trauma, intraventricular hemorrhage, seizure at less than 24 hours, invasive and non-invasive mechanical ventilation, necrotizing enterocolitis, hypoxic-ischemic encephalopathy, cardiopulmonary morbidity, neonatal infection/ sepsis, blood transfusion.

Table S8: Intraclass correlation (ICC) for costs and effects[a]

Crude Model	Intraclass correlation (ICC)	Category[a]
Effects (cesarean sections)	0.006	Low
Costs	0.020	Moderate

Adjusted Model	Intraclass correlation (ICC)	Category[a]
Effects (cesarean sections)	0.008	Low
Costs	0.015	Moderate

[1] Based on bivariate multilevel linear models for the main analysis presented in Table 1

[2] We classified the intraclass correlation (ICC) for costs and outcomes as "high" (ICC > 0.1), "moderate" (0.01 < ICC ≤ 0.1), or "low" (ICC ≤ 0.01).[1]

Table S9: Individual-level correlation between costs and effects[1]

	Model 1 (Crude)	Model 2 (Adjusted)
**All participants**	0.263	0.235
**Low-risk subgroup**	0.239	0.208
**High-risk subgroup**	0.236	0.242

[1] Based on bivariate multilevel linear models for the main analysis presented in Table 1

Table S10: Return on Investment

Investment		Earnings	
Per capita hospital costs	$89,455	**Per capita saving per woman**	$180 (95%CI: $83 to $277)
# intervention hospitals	16	**# IG deliveries in postintervention period**	23,484
	$1,431,280		$4,227,120 ($1,949,172 to $6,505,068)
		Return on Investment (Year 1)	$2,795,840 ($517,892 to 5,073,788)

References

1. Gomes M, Grieve R, Nixon R, Edmunds WJ. Statistical methods for cost-effectiveness analyses that use data from cluster randomized trials: a systematic review and checklist for critical appraisal. Med Decis Making 2012;32:209-20.
2. Gomes M, Ng ES, Grieve R, Nixon R, Carpenter J, Thompson SG. Developing appropriate methods for cost-effectiveness analysis of cluster randomized trials. Med Decis Making 2012;32:350-61.
3. Manning WG, Mullahy J. Estimating log models: to transform or not to transform? Journal of health economics 2001;20:461-94.
4. Rasbash J, Charlton C, Browne WJ, Healy M, Cameron B. MLwiN Version 2.1. Centre for Multilevel Modelling, University of Bristol; 2009.
5. Leckie G, Charlton C. runmlwin: A program to run the MLwiN multilevel modeling software from within Stata. 2013 2013;52:40.
6. Canadian Institute for Health Information (CIHI). National Physician Database 2011-2012 Data Release2013. Tables Table B.7.3: Surgical Specialists—Cost per Service by National Grouping System Category and Province/Territory, 2011–2012
7. Canadian Institute for Health Information (CIHI). Patient cost estimator methodology notes and glossary. Ottawa: CIHI; 2015:9.
8. Chaillet N, Dumont A, Abrahamowicz M, et al. A cluster-randomized trial to reduce cesarean delivery rates in Quebec. N Engl J Med 2015;372:1710-21.
9. Patient cost estimator CIHI, 2015. at https://www.cihi.ca/en/spending-and-health-workforce/spending/patient-cost-estimator.)
10. Guidance document for the costing of health care resources in the Canadian setting: 2nd edition. Ottawa2016 March.
11. Canadian Institute for Health Information (CIHI). Per diem costs (ward & ICU) for acute care hospitals in the Province of Ontario, 2011-2012. Ottawa: CIHI; 2016.
12. Wunsch H, Angus DC, Harrison DA, Linde-Zwirble WT, Rowan KM. Comparison of medical admissions to intensive care units in the United States and United Kingdom. Am J Respir Crit Care Med 2011;183:1666-73.

13. Inflation Calculator Government of Canada, 2016. (Accessed 2016-08-08, 2016, at http://www.bankofcanada.ca/rates/related/inflation-calculator/.)

14. Régie de l'assurance maladie du Québec. Manuel de facturation: Rémunération à l'acte (médecins spécialistes). MAJ 85 ed2013.

15. Exchange Rates. Government of Canada, 2013. (Accessed 2015-10-30, 2015, at http://www.bankofcanada.ca/rates/exchange/.)
